# Folylpolyglutamate synthase is a major determinant of intracellular methotrexate polyglutamates in patients with rheumatoid arthritis

**DOI:** 10.1038/srep35615

**Published:** 2016-10-18

**Authors:** Tatsuhiro Yamamoto, Kotaro Shikano, Toshihiro Nanki, Shinichi Kawai

**Affiliations:** 1 Division of Rheumatology, Department of Internal Medicine, School of Medicine, Faculty of Medicine, Toho University, Tokyo 143–8541, Japan.

## Abstract

We investigated major determinants of the intracellular concentrations of methotrexate polyglutamates (MTXPGs) in patients with rheumatoid arthritis (RA). In 271 RA patients on stable oral low dose weekly pulse MTX therapy, the concentrations of MTXPGs in red blood cells (RBCs) were measured by liquid chromatography-electrospray ionization-tandem mass spectrometry. Polymerase chain reaction-restriction fragment length polymorphism analysis was performed to determine the genotypes of *solute carrier family 19 member 1* (*SLC19A1*), *folylpolyglutamate synthase* (*FPGS*), and *gamma-glutamyl hydrolase* (*GGH*). The mean total MTXPG concentration and the concentrations of individual MTXPGs increased dose-dependently, but reached a plateau at MTX doses >10 mg weekly. The MTXPG3-5/1-2 ratio was lower in patients with adverse events related to MTX than in patients without adverse events. Three polymorphisms of *FPGS* significantly influenced the MTXPG3-5/1-2 ratio in RBCs, while polymorphisms of *SLC19A1* and *GGH* had no impact. The minor allele frequencies of 2 *FPGS* genotypes were significantly increased in our patients compared with a Caucasian population. FPGS may have a major role in regulating intracellular polyglutamation of MTX in RA patients receiving low-dose weekly MTX therapy.

Methotrexate (MTX) is recommended as first-line therapy in the recent American^1^, European^2^, and Japanese^3^ recommendations and/or guidelines regarding the treatment of rheumatoid arthritis (RA). MTX is the most frequently used disease-modifying antirheumatic drug (DMARD), and is essential for treatment of RA as both monotherapy and combined with low molecular weight or biologic DMARDs. There is substantial evidence regarding the efficacy of MTX for RA, but the response to this drug is known to vary among patients.

MTX binds to a folate transporter (solute carrier family 19, member 1 [SLC19A1], also known as reduced folate carrier 1) in order to enter target cells^4^. Inside the cells, folylpolyglutamate synthetase (FPGS) converts MTX into MTX polyglutamates (MTXPGs), which show long-term persistence in target cells. Gamma-glutamyl hydrolase (GGH) is involved in removing glutamates from MTXPGs. After MTXPGs are converted back to MTX by GGH, the drug is removed from cells by adenosine triphosphate (ATP)-binding cassette transporters.

MTXPGs have a higher binding affinity for dihydrofolate reductase (DHFR) than MTX, and binding with MTXPGs inhibits DHFR activity^5^ to suppress tetrahydrofolate production. Tetrahydrofolate is required for DNA synthesis and plays a vital role as an essential coenzyme in various aspects of amino acid metabolism, such as serine–glycine conversion and methionine synthesis. MTXPGs also inhibit aminoimidazole carboxamide ribonucleotide transformylase (ATIC), causing the intracellular accumulation of aminoimidazole carboxamide ribonucleotide, which in turn inhibits adenosine-metabolizing enzymes. Additionally, intracellular MTXPGs are known to show greater binding affinity for ATIC^6^ compared with MTX. Therefore, intracellular MTXPGs are considered to have a major role in suppressing both cell-mediated immunity and humoral immunity, as well as exerting anti-inflammatory and immunosuppressive effects through inhibition of DNA synthesis or amino acid metabolism.

The treatment regimen of MTX for RA is low-dose weekly pulse administration in global guidelines^1^,^2^,^3^. Since the efficacy of therapy for RA is maintained during several weeks, the half-life of MTX in the blood which was reported to be 4.9–7.3 hours^7^,^8^ does not directly reflect the therapeutic efficacy in RA patients^8^. For that reason and because of its known mechanism of action, attention has been directed toward the intracellular MTXPG concentration as a potential predictor of the response to MTX treatment.

In general, the cells that are considered to be direct targets of MTX (such as peripheral blood lymphocytes) are difficult to collect an adequate quantity, and therefore measurement of the MTX concentration in these cells is not easy in human studies^9^. Instead, researchers have measured the MTXPG concentration in red blood cells (RBCs) and have investigated its association with the clinical response to MTX^9^,^10^. In the present study, we used liquid chromatography-tandem mass spectrometry (LC-MS/MS) to measure the concentrations of individual MTXPGs in RBCs and explored the association of each MTX derivative with clinical indicators. We also investigated polymorphism of the *SLC19A1*, *FPGS*, and *GGH* genes, which play a vital role in intracellular metabolism of MTX.

## Results

### Concentrations of MTXPGs

**Figure 1:**
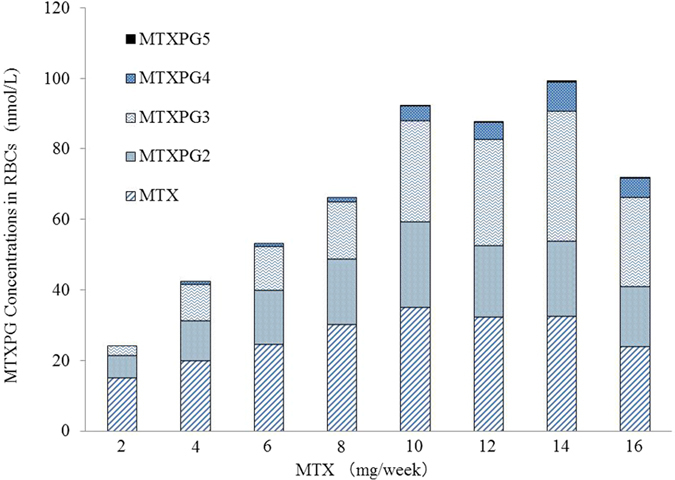
Concentrations of methotrexate polyglutamates in red blood cells of patients with rheumatoid arthritis on methotrexate therapy. MTX = methotrexate, PG = polyglutamate, RBC = red blood cells.

MTX has 1 glutamate moiety and is thus referred to as MTXPG1. Figure 1 shows the concentrations of MTXPG1, MTXPG2, MTXPG3, MTXPG4, and MTXPG5 in RBCs harvested from patients with RA categorized by the weekly MTX dose. The mean total MTXPG concentration and the concentrations of individual MTXPGs increased dose-dependently. However, concentrations of the MTXPGs were nearly constant in patients receiving MTX at doses of equal and more than 10 mg per week. MTXPG6 was not detected in any of the samples.

### Adverse events

We used criteria of the adverse events which were defined by the Japanese Pharmaceuticals and Medical Devices Agency. As shown in Table 1, the average dose of MTX administered in the AE (−) group was 9.5 mg/week, which was significantly higher than the average dose of the AE (+) group (6.9 mg/week). There was no significant difference of RA disease activity (Disease Activity Score of 28 joints-erythrocyte sedimentation rate (DAS28-ESR)^11^, Clinical Disease Activity Index (CDAI)^12^, Simplified Disease Activity Index (SDAI)^13^) between the AE (−) and AE (+) groups. However, a significantly higher percentage of patients received other DMARDs and the biological agents in the AE (+) group than in the AE (−) group.

The following adverse events were observed in the AE (+) group: elevation of liver enzymes (68 events), dyspepsia and/or oral aphthous ulcers (12 events), decreased white blood cell count (15 events), and others (5 events). None of these events were severe and they improved with reduction of the MTX dose, which was subsequently maintained for at least more than 3 months before collection of blood samples.

**Figure 2:**
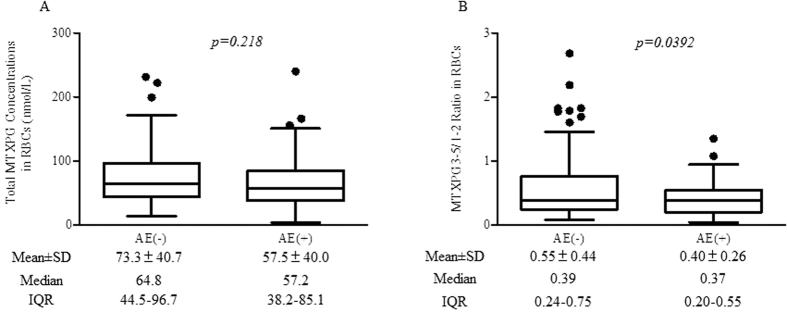
Total methotrexate concentration and MTXPG3-5/1-2 ratio in red blood cells of rheumatoid arthritis patients with or without adverse events during methotrexate therapy. (**A**) Total methotrexate concentration. (**B**) MTXPG3-5/1-2 ratio.

When the RBC concentration of MTXPG was evaluated in the AE (−) and AE (+) groups, there was no significant difference between the two groups (*p* = 0.218, Fig. 2A). In contrast, the RBC MTXPG3-5/1-2 ratio was significantly lower in the AE (+) group than in the AE (−) group (*p* = 0.036, Fig. 2B).

### Correlation between MTXPG concentration and clinical parameters

Correlations between RBC concentrations of total MTXPGs and variable clinical parameters (sex, disease duration, age, disease progression category (stage), and DAS28-ESR) in patients with RA receiving MTX therapy were evaluated by univariate and multivariate analyses (Table 2). Associations between the total MTXPG concentrations in RBCs with clinical parameters in RA patients were not significant with various parameters except for dose of MTX in both analyses (p < 0.000).

### Polymorphism of *SLC19A1*, *FPGS,* and *GGH*

To examine the association between the MTXPG concentration in RBC and polymorphism of the genes for enzymes involved in intracellular MTX metabolism, we investigated polymorphisms of *SLC19A1*, *FPGS*, and *GGH* (Table 3). The total intracellular MTX concentration and the levels of MTXPG1-2 and MTXPG3-5 were not significantly different among the 3 genotypes for a SNP of *SLC19A1* or for 5 SNPs of *GGH* (data not shown). The MTXPG3-5/1-2 ratio also showed no significant difference among the 3 genotypes for the SNPs of *SLC19A1* or *GGH*. Although the total intracellular MTXPG1-5 concentration and the concentrations of MTXPG1-2 and MTXPG3-5 were not significantly different among the 3 genotypes for 3 SNPs of *FPGS* (data not shown), the MTXPG3-5/1-2 ratio showed significant differences among these 3 genotypes for 3 different SNPs of *FPGS* (Table 3). These results indicate that, among the enzymes involved in intracellular conversion of MTX to MTXPGs, polymorphisms of the *FPGS* gene are determinants of the intracellular levels of MTXPGs.

We also examined the relations between polymorphisms of *SLC19A1*, *FPGS,* or *GGH* and adverse events, but there were no significant differences in the distribution of *SLC19A1*, *FPGS,* and *GGH* genotypes between the AE (−) and AE (+) groups (Table 4).

### Allele frequencies of *SLC19A1*, *FPGS,* and *GGH*

Table 5 shows the allele frequencies of several *SLC19A1*, *FPGS,* and *GGH* genotypes in our RA patients and in the general Japanese and Caucasian populations [cited from the International HapMap project (http://hapmap.ncbi.nlm.nih.gov/)]. There were no significant differences in the allele frequencies of a SNP of *SLC19A1* (rs1051266) and 3 SNPs of *GGH* (rs1800909, rs3758149, and rs11545078) between our patients and the Japanese population (HapMap project) (data not shown). However, the distributions of 2 SNPs of *FPGS* (rs10106 and rs154410) and 2 SNPs of *GGH* (rs719235 and rs12681874) showed significant differences between Caucasians and our patients.

## Discussion

Because this study was performed in the clinical practice setting experience, the weekly dose of MTX was determined by the attending physician of each patient. Therefore, the total MTXPG concentration in RBCs and the individual concentrations of MTXPG1-5 were analyzed after stratification by the weekly dose. This analysis revealed that the MTXPG concentration increased in a dose-dependent manner, but almost showed a plateau in patients administered equal and more than 10 mg of MTX weekly. The small changes of MTXPG concentrations in our patients on higher MTX doses were similar to the results reported by Stamp^14^, who found stable MTXPG levels at MTX doses more than 15 mg weekly.

Direct targets of MTX therapy are supposed as immune-related cells such as lymphocytes. In fact, lymphocyte counts are approximately 2,000/μL, while RBC counts are 4 million/μL of whole blood. We used 6 mL of whole blood to measure the concentration of MTXPGs in which almost all cellular components are RBCs. If we measure the MTXPGs in lymphocytes using our system, we need approximately 12 L of whole blood. From ethical point of view, we measured the MTXPGs in RBCs as a surrogate marker represented with the immune-related cells.

The MTXPG3-5/1-2 ratio at an MTX dose of 10 mg weekly was 0.639 in our study, whereas it was more than 1.0 according to Stamp, which is considerably higher than in the present investigation. There was no difference in the duration of MTX treatment (5.8 vs. 4.5 years, respectively) between the two studies. Since MTXPG3-5/1-2 is affected by the enzymatic potencies of glutamation (FPGS) and deglutamation (GGH) for MTX and/or MTXPGs, it is suspected that polymorphism of genes encoding enzymes related to metabolism of MTX might explain the differences of polyglutamation in these different ethnic cohorts.

In this study, we found that the MTXPG3-5/1-2 ratio was significantly influenced by 3 different SNPs of *FPGS*, but we did not find any relationship between the MTXPG3-5/1-2 ratio and SNPs of *SLC19A1* or *GGH*. These results strongly suggested that FPGS enzyme activity, rather than GGH activity or SLC19A1 transport function, was a major determinant of intracellular conversion of MTX to MTXPG3-5.

In patients with acute leukemia, it was reported that the activity of GGH and FPGS in blast cells is a good predictor of the relative levels of MTXPG3-5, with lower FPGS and/or higher GGH activity leading to reduced MTX polyglutamation^15^. In addition, the mutation Cys346Phe of *FPGS* gene reduces enzyme activity in several human leukemia cell lines^16^.

As shown in Table 5, there were significant differences in the minor allele frequencies of 2 SNPs of *FPGS* (rs10106 and rs1544105) between our Japanese RA patients and the general Caucasian population. Since the MTXPG3-5/1-2 ratio was higher in our patients with 1994GG, 11694TT, 2572TT of *FPGS* compared to other genotypes, respectively (Table 3), the increased frequency of 1994GG and 2572TT of *FPGS* in Caucasians might have been associated with higher FPGS activity and possively with the elevation of the MTXPG3-5/1-2 ratio. In addition, we cited the minor allele frequencies of Japanese general populations from HapMap project (Table 5). There was no significant difference in minor allele frequencies of *SLC19A1*, *FPGS* and *GGH* between our Japanese RA patients and Japanese general populations. Although we do not have the data of these allele frequencies in Caucasian RA patients, it is suggested that these genotypes might not be influenced by RA itself.

The average weekly dose of MTX was 8.7 mg in our study, while the average weekly dose of MTX was reported as 5.2 and 7.2 mg in other Japanese RA cohorts^17^,^18^. However, the average weekly dose of MTX was 15 mg in studies from the USA^19^ and the Netherlands^20^. While part of the difference in MTX doses is probably attributable to differences of body weight, the differing minor allele frequencies of *FPGS* polymorphisms might also be a contributing factor for the higher tolerability of MTX therapy in Caucasians.

It has been reported that higher MTXPG4-5 concentrations were significantly associated with adverse events in patients with inflammatory bowel disease receiving MTX^21^. In patients with juvenile idiopathic arthritis, MTXPG3–5 concentrations were positively correlated with elevation of liver function parameters and gastrointestinal adverse events^22^. On the contrary, other studies conducted in RA patients^14^,^19^ showed no association between MTXPG concentrations and adverse events. We also found that the concentrations of MTXPGs in RBCs did not related with disease activities and did not differ between the AE (−) and AE (+) groups. Since our present study is a cross-sectional examination which might be influenced by many other factors such as other therapeutic agents, we could not conclude whether the measurement of the intracellular MTXPGs was a proper index for therapeutics or not. We then focused to the detailed mechanisms of the intracellular metabolism of MTX. To clarify the relationship between MTXPG concentrations and efficacy or safety of MTX, a prospective study in the RA patients who were started with MTX therapy will be necessary.

We found that the MTXPG3-5/1-2 ratio decreased significantly in the AE (+) patients in whom dose of MTX was reduced by the attending physician due to the non-severe adverse events. Thus, it could be suggested that relatively high MTXPG1-2 levels might be a contributor to dose-dependent non-severe adverse events. MTXPGs inhibit DHFR and ATIC more strongly than MTX itself 5,6, suggesting a major role in the actions of MTX, but the mechanisms underlying dose-dependent adverse events have not been clarified yet. The results of the present study suggested that reduced polyglutamation of MTX might be associated with a higher incidence of adverse events.

Many previous studies have explored how polymorphisms of MTX target molecules are related to the treatment response and adverse events. Polymorphisms of the *methylenetetrahydrofolate reductase* and *ATIC* genes have been suggested to have an important influence on the clinical response to MTX^23^, but our study focused on intracellular MTX metabolism so we did not investigate these SNPs. It is possible that combined assessment of the polymorphisms of target molecules and enzymes involved in MTX metabolism might provide more useful information for clinical management of MTX therapy.

## Methods

### Patients

The subjects were 271 patients with RA on stable oral MTX pulse therapy at Toho University Omori Medical Center Hospital (Table 1). Only patients who met the 2010 American College of Rheumatology/European League Against Rheumatism criteria for rheumatoid arthritis were included in this study. Among them, 188 patients had no adverse events at the MTX dose being administered at the time of blood collection and they were assigned to the AE (−) group. We used criteria of the adverse events which were defined by the Japanese Pharmaceuticals and Medical Devices Agency for evaluation of adverse events in our study. All patients received a stable dose of MTX orally for at least more than 3 months before blood sample collection. They all took folic acid (5 mg/week) at 48 hours after MTX administration.

Demographic and clinical details were collected by using standard data collection forms. Disease activity was assessed from the swollen joint count, tender joint count, and physician’s global assessment of disease activity, and patient’s assessment of pain and global assessment of disease activity, which were measured by 100 mm visual analog scales (VAS). DAS28-ESR (0.56 × √(tender joint count) + 0.28 × √(swollen joint count) + 0.7 × LN(ESR) + 0.014 × (VAS))^11^ was calculated, as were CDAI (tender joint count + swollen joint count + patient’s VAS + physician’s VAS)^12^ and SDAI (CDAI + C-reactive protein)^13^.

The protocol of this study was approved by the Institutional Review Board for Genetic Research of Toho University (approval number: 24-2) and written informed consent was obtained from all of the patients prior to enrollment. This study was carried out in accordance with Ethical Guidelines for Human Genome/Gene Analysis Research by Ministries of Education, Culture, Sports, Science and Technology; Health, Labour and Welfare; and Economy, Trade and Industry of the Japanese Government.

### Determination of MTXPG in RBCs

Whole blood sample was drawn at various timing from the day of oral MTX administration, however, no patient received MTX just the same day of the sampling. A sample of whole blood (6 ml) was collected in a tube containing EDTA. After removing 0.3 mL of whole blood for genetic testing, RBCs and plasma were immediately separated by centrifugation for 10 min, and then were stored at −80 °C until analysis.

MTXPGs were measured in aliquots of the cell pellet with an LC-MS/MS assay system by the method of den Boer *et al*.^24^ with slight modifications. Briefly, analyses were performed on a LC-MS/MS system consisting of a UFLC/TSQ Quantum Ultra (Shimadzu, Kyoto, Japan) triple quadrupole mass spectrometer with an electrospray ionization source. MTXPG1-6 standards were purchased from Schircks Laboratories (Jona, Switzerland). LC-MS-grade methanol, ammonium bicarbonate, and perchloric acid were obtained from Wako Junyaku (Tokyo, Japan), while ammonium hydroxide was purchased from Sigma-Aldrich (St. Louis, MI). Chromatography was performed after partial-loop injection of a 10 μL sample, using a YMC-Triart C18 column (2.0 × 100 mm, 3.0 μm) (YMC, Kyoto, Japan) at 40 °C. The mobile phase consisted of (A) 10 mM ammonium bicarbonate adjusted to pH 10 with 25% ammonium hydroxide and (B) acetonitrile at a flow rate of 0.4 mL/min. Elution was done according to the following program: isocratic hold with 2% B for 0–4.5 min, a linear gradient to 40% B from 4.5–5.1 min; a linear gradient to 95% B from 5.1–6.1 min; and isocratic hold with 98% B from 6.1–11.00 min. The electrospray ionization source was operated in the positive mode with the following fixed settings and capillary voltage, 1.00 kV; dissolution temperature of 220 °C, nitrogen gas flow rate of 1,000 L/hour, and cone gas (nitrogen) flow rate of 50 L/hour. Argon was used as the collision gas at a flow rate of 0.20 mL/min. The specific settings for each MTXPG were as follows: dwell time was 0.1 second for all MTXPGs, while the cone voltage was 30, 30, 50, 60, 55, and 55 V for MTXPG1 to MTXPG6, respectively. Collision energy was set at 20, 20, 40, 40, 50, and 50 eV for MTXPG1 to MTXPG 6, respectively.

### Genetic analysis

Samples of whole blood (0.3 mL) were stored at −80 °C until DNA was extracted with a Maxwell 16 DNA Purification Kit (Promega, IL). Then single nucleotide polymorphisms (SNPs) of the *SLC19A1* (rs1051266), *FPGS* (rs10106, rs1054774, rs1544105), and *GGH* (rs719235, rs1800909, rs3758149, rs11545078, rs12681874) genes were determined by the polymerase chain reaction-restriction fragment length polymorphism assay using a TaqMan kit (Applied Biosystems, Foster City, CA) according to the manufacturer’s protocol.

### Statistical analysis

Stastical analysis was performed with Prism ver. 5.0 software (Graphpad Software, San Diego, CA). Numerical data are expressed as both the mean ± SD and the median with interquartile range (IQR). The Mann-Whitney U test and the chi-square test (or Fisher’s exact test) were used to compare categorical data between two groups, while the Kruskal-Wallis test was applied to compare numerical data among three groups. Simple linear regression analysis was used to assess correlations between RBC MTXPGs concentrations and patient characteristics. Stepwise forward multiple regression analysis was also performed. The level of significance was set at P < 0.05 for all analyses.

## Additional Information

**How to cite this article**: Yamamoto, T. *et al*. Folylpolyglutamate synthase is a major determinant of intracellular methotrexate polyglutamates in patients with rheumatoid arthritis. *Sci. Rep.* **6**, 35615; doi: 10.1038/srep35615 (2016).

## Figures and Tables

**Table 1:** Demographic profile of the study population.

	(n = 271)	AE (−) group	AE (+) group	P value
		(n = 188)	(n = 83)	
Age (years)	60.4 ± 12.6	60.0 ± 13.2	61.4 ± 13.8	*P* = *0.464*
No. of men/women	59/212	48/140	11/72	*P* = *0.031*
Duration (months)	107.3 ± 96.0	103.0 ± 93.9	117.0 ± 98.2	*P* = *0.176*
Dose of MTX (mg/week)	8.7 ± 3.1	9.5 ± 3.0	6.9 ± 2.5	*P* < *0.001*
Stage (I/II/III/IV)	(90/61/46/74)	(68/42/31/47)	(22/19/15/27)	
CRP (mg/dl)	0.22 ± 0.13	0.23 ± 0.35	0.19 ± 0.22	*P* = *0.942*
ESR (mm/hour)	15.8 ± 13.3	15.4 ± 13.0	16.5 ± 15.6	*P* = *0.671*
DAS28-ESR	2.29 ± 1.05	2.35 ± 1.09	2.17 ± 0.95	*P* = *0.227*
CDAI	3.30 ± 4.26	3.80 ± 5.03	3.20 ± 4.88	*P* = *0.618*
SDAI	5.51 ± 5.27	5.12 ± 6.74	5.14 ± 5.45	*P* = *0.881*
BMI	22.0 ± 3.4	21.6 ± 3.6	22.6 ± 3.9	*P* = *0.889*
BW(kg)	55.5 ± 11.2	56.4 ± 9.2	53.4 ± 10.8	*P* = *0.926*
No. of patients using: Prednisolone	83(30.6%)	53(28.2%)	30(36.1%)	*P* = *0.195*
Other DMARDs	51(18.8%)	24(12.8%)	27(32.5%)	*P* < *0.001*
Biological agents	134(49.4%)	82(43.6%)	52(62.7%)	*P* < *0.001*

Data are the mean±SD, n  =  number of patients

*P values* for comparison between the AE (−) and AE (+) groups were evaluated by the Mann-Whitney U test and Fisher’s exact test.

MTX: methotrexate, stage: Steinblocker’s classification of progression, CRP: C-reactive protein, ESR: erythrocyte sedimentation rate, DAS: disease activity score, CDAI: clinical disease activity index, SDAI: simplified disease activity index, BMI: body mass index, DMARDs: disease-modifying anti-rheumatic drugs.

**Table 2:** Correlations between RBC concentrations of total MTXPGs and variable clinical parameters in patients with RA receiving MTX therapy.

	Total MTXPGs				
			Univariate model				Multivariate model		
	β	*p* value	R^2^	β	*p* value			
Sex	0.019	0.761	0.019	−0.014	0.822
Duration	0.012	0.842	0.012	0.127	0.095
Dose of methotrexate	0.399	0.000	0.156	0.421	0.000
Age	0.102	0.099	0.007	0.162	0.081
Stage	0.102	0.112	0.112	−0.019	0.802
DAS28-ESR	0.091	0.147	0.004	0.049	0.432
R^2^				0.180	

β: regression coefficient; Stage; Steinblocker’s classification of progression; DAS28-ESR: disease activity score-erythrocyte sedimentation rate; R^2^: coefficient of determination.

**Table 3:** Relationship between Polymorphisms of *SCL19A1*, *FPGS*, or *GGH* and the Intracellular MTXPG3-5/1-2 Ratio.

SNP	rs No.	genotype	n	Intracellular MTXPG3-5/1-2 ratio		P value
				Mean ± SD	Median [IQR]	
*SLC19A1*	rs1051266	GG	94	0.479 ± 0.356	0.367 [0.250–0.607]	*p* = *0.475*
		AG	130	0.556 ± 0.466	0.427 [0.234–0.794]	
		AA	47	0.445 ± 0.334	0.338 [0.157–0.707]	
*FPGS*	rs10106	AA	130	0.457 ± 0.375	0.345 [0.201–0.567]	*p* = *0.006*
		AG	115	0.503 ± 0.394	0.396 [0.235–0.656]	
		GG	26	0.714 ± 0.465	0.672 [0.364–0.957]	
	rs1054774	TT	113	0.555 ± 0.404	0.428 [0.264–0.788]	*p* = *0.008*
		AT	122	0.463 ± 0.396	0.350 [0.201–0.562]	
		AA	36	0.417 ± 0.362	0.319 [0.161–0.568]	
	rs1544105	CC	128	0.454 ± 0.372	0.347 [0.202–0.565]	*p* = *0.003*
		CT	120	0.506 ± 0.391	0.396 [0.235–0.700]	
		TT	23	0.747 ± 0.489	0.634 [0.385–1.030]	
*GGH*	rs719235	CC	216	0.501 ± 0.389	0.385 [0.238–0.652]	*p* = *0.862*
		CT	47	0.486 ± 0.378	0.385 [0.184–0.633]	
		TT	8	0.645 ± 0.702	0.382 [0.205–0.859]	
	rs1800909	CC	223	0.495 ± 0.397	0.397 [0.220–0.634]	*p* = *0.882*
		CT	36	0.524 ± 0.401	0.255 [0.255–0.668]	
		TT	12	0.444 ± 0.229	0.375 [0.297–0.710]	
	rs3758149	GG	232	0.498 ± 0.401	0.383 [0.234–0.616]	*p* = *0.889*
		GT	27	0.552 ± 0.422	0.381 [0.240–0.869]	
		TT	12	0.441 ± 0.241	0.385 [0.274–0.697]	
	rs11545078	TT	233	0.495 ± 0.391	0.376 [0.234–0.634]	*p* = *0.321*
		CT	37	0.558 ± 0.437	0.430 [0.232–0.702]	
		CC	1	0.151	0.151	
	rs12681874	GG	81	0.452 ± 0.332	0.362 [0.235–0.604]	*p* = *0.070*
		AG	134	0.600 ± 0.472	0.442 [0.254–0.812]	
		AA	60	0.493 ± 0.418	0.345 [0.197–0.687]	

n  =  number of patients

*p* values for comparisons among 3 groups were evaluated by the Kruskal-Wallis test.

*SLC19A1: reduced folate carrier 1, FPGS: folylpolyglutamyl synthase, GGH: gamma-glutamyl hydrolase.*

**Table 4:** Relationships between Polymorphisms of *SLC19A1, FPGS,* or *GGH* and adverse events stratified by genotype.

		genotype	AE(−) group	AE (+) group	P value
*SLC19A1*	rs1051266	80G > A	GG/AG/AA	GG/AG/AA	*p* = *0.139*
			68/93/27	26/37/20	
*FPGS*	rs10106	1994A > G	AA/AG/GG	AA/AG/GG	*p* = *0.952*
			91/78/19	39/37/7	
	rs1054774	11694T > A	TT/AT/AA	TT/AT/AA	*p* = *0.289*
			73/89/26	40/38/10	
	rs1544105	2572C > T	CC/CT/TT	CC/CT/TT	*p* = *0.327*
			93/80/15	35/40/8	
*GGH*	rs719235	452C > T	CC/CT/TT	CC/CT/TT	*p* = *0.688*
			151/32/5	65/15/3	
	rs1800909	−401C > T	CC/CT/TT	CC/CT/TT	*p* = *0.477*
			7/22/159	5/14/64	
	rs3758149	−354G > T	GG/GT/TT	GG/GT/TT	*p* = *0.125*
			121/61/6	56/23/4	
	rs11545078	16T > C	TT/CT/CC	TT/CT/CC	*p* = *0.582*
			163/25/0	70/12/1	
	rs12681874	14269G > A	GG/AG/AA	GG/AG/AA	*p* = *0.174*
			61/92/39	20/42/21	

*p values* for comparisons between the AE (+) and AE (−) groups were evaluated by the Kruskal-Wallis test.

*SLC19A1: reduced folate carrier 1, FPGS: folylpolyglutamyl synthase, GGH: gamma-glutamyl hydrolase.*

**Table 5:** Allele frequency in several genotypes of *SLC19A1 FPGS* and *GGH* in our patients with rheumatoid arthritis and in general populations of Japanese and Caucasian.

SNPs	No of rs	genotype	Minor Allele Frequency			P value
			Our study	Japanese	Caucasian	
*SLC19A1*	rs1051266	80G > A	0.698/0.302	0.512/0.488	0.438/0.562	*p* = 0.210
*FPGS*	rs10106	1994A > G	0.693/0.307	0.727/0.273	0.450/0.550	*p* < 0.01
	rs1054774	11694T > A	0.360/0.640	0.330/0.670	0.342/0.658	*p* = 0.471
	rs1544105	2572C > T	0.717/0.283	0.727/0.273	0.342/0.658	*p* < 0.01
*GGH*	rs719235	452C > T	0.892/0.108	0.895/0.105	0.677/0.323	*p* < 0.01
	rs1800909	−401C > T	0.850/0.150	0.727/0.273	0.342/0.658	*p* = 0.478
	rs3758149	−354G > T	0.799/0.201	0.640/0.360	0.692/0.308	*p* = 0.549
	rs11545078	16T > C	0.952/0.048	0.856/0.144	0.925/0.075	*p* = 1
	rs12681874	14269G > A	0.638/0.362	0.533/0.467	0.890/0.110	*p* < 0.01

^*^p values between our patients and Caucasian data were analyzed by Fisher’s test.

*SLC19A1*: reduced folate carrier 1, *FPGS*: folylpolyglutamyl synthase, *GGH*: gamma-glutamyl hydrolase.
